# Butylene Glycol Used as a Sustainable Solvent for Extracting Bioactive Compounds from *Camellia sinensis* Flowers with Ultrasound-Assisted Extraction

**DOI:** 10.1021/acsomega.2c07481

**Published:** 2023-01-25

**Authors:** Hla Myo, Nara Yaowiwat, Pawin Pongkorpsakol, Chanat Aonbangkhen, Nuntawat Khat-udomkiri

**Affiliations:** † School of Cosmetic Science, Mae Fah Laung University, Chiang Rai57100, Thailand; ‡ Princess Srisavangavadhana College of Medicine, Chulabhorn Royal Academy, Bangkok10210, Thailand; § Department of Chemistry, Faculty of Science, Chulalongkorn University, Bangkok10330, Thailand; ∥ Center of Excellence in Natural Products Chemistry (CENP), Department of Chemistry, Faculty of Science, Chulalongkorn University, Bangkok10330, Thailand

## Abstract

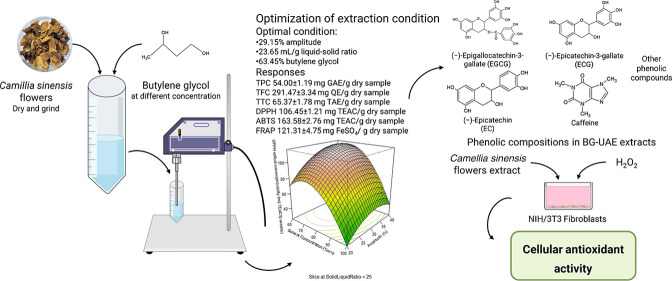

The research aims to assess the yield of bioactive compounds and their antioxidant activities obtained from tea flowers using an ultrasound-assisted extraction method with butylene glycol (BG-UAE) through Box–Behnken design. It investigates the bioactive compounds including the total phenolic content (TPC), total flavonoid content (TFC), and total tannin content (TTC) and analyzes their antioxidant activities, bioactive compound composition by liquid chromatography triple quadrupole tandem mass spectrometry, and their cellular activities via UAE and maceration using BG or ethanol as the solvent. Under optimal conditions, the values of the TPC, TFC, TTC, 1,1-diphenyl-2-picrylhydrazil radical scavenging assay, 2,2′-azino-bis-3-ethylbenzothiazoline-6-sulphonic acid radical scavenging assay, and ferric reducing antioxidant power assay (FRAP) of the BG-UAE extract were 54.00 ± 1.19 mg GAE/g sample, 291.47 ± 3.34 mg QE/g sample, 65.37 ± 1.78 mg TAE/g sample, 106.45 ± 1.21 mg TEAC/g sample, 163.58 ± 2.76 mg TEAC/g sample, and 121.31 ± 4.75 mg FeSO_4_/g sample, respectively. Except for FRAP, BG-UAE exhibited the highest values in all parameters compared to the other extraction methods. Catechins and caffeine were predominantly detected in tea flower extracts through UAE with BG and ethanol (EtOH-UAE). BG-UAE exhibited greater cell viability and cellular antioxidant activity than EtOH-UAE. The researcher expects that this research will contribute to the emergence of a green extraction technique that will offer larger functional components with economic and environmental benefits and minimal chemicals and energy use.

## 1 Introduction

Secondary metabolites, also known as “phytochemicals” or “bioactive compounds”, are extracted from agricultural biomass and comprise a wide range of natural products, including phenols, flavonoids, tannins, coumarins, terpenoids, alkaloids, saponins, quinones, glycosides, and steroids.^1^ The most abundant phytochemicals obtained from edible plants (agricultural biomass) are phenolic compounds (including flavonoids) which play a crucial role in non-enzymatic antioxidant activities, protection of cells from oxidative stress damage, health promotion, and disease prevention.^1,2^

Nowadays, substantial emphasis has been placed on reducing or eliminating the use of organic solvents that have detrimental effects on human health and the environment along with increased public sentiment in protecting the environment.^3^ Previously, most extraction procedures in cosmetic and pharmaceutical industries relied largely on petroleum-based solvents, which have been linked to adverse health and environmental effects. Therefore, the search for a novel solvent that is environmental friendly to replace conventional organic solvents has evolved into a growing subject of research in the development of green extraction.^3^

Organic solvents used in the extraction of bioactive compounds from different plants need to be separated from the extracts due to the potential hazards of their residuals to human skin.^4^ This separation procedure takes time, is costly, and consumes much energy. To tackle this challenge, butylene glycol (BG) could be an alternative solvent for plant extracts, as it can be used in cosmetic and pharmaceutical preparations without being removed from the extract and is categorized as a generally recognized as safe chemical by the United States Food and Drug Administration (FDA). In addition, BG is being used as a solvent for extracting bioactive compounds from various parts of plants including *Camellia* seed dregs,^5^*Camellia japonica* leaf,^6^ and apple waste peel.^7^

Among the various extraction techniques, ultrasound-assisted extraction (UAE) is considered a green extraction method. UAE uses ultrasound waves to exert thermal and mechanical effects and cavitation on cell walls or tissues to release bioactive components into the extraction solvent. It is known as an eco-friendly extraction method that has a minimal environmental impact as the calculated carbon dioxide rejection rates of UAE are lower than those of Soxhlet extraction and maceration.^8^ In addition, the bioactive molecules obtained from plants by UAE show better quality, higher yield, and less degradation than those obtained using conventional techniques.^9^

Tea (*Camellia sinensis*) is widely cultivated in over 30 countries including Thailand, and it can grow well in tropical and subtropical regions.^10^ The parts of tea plants widely used are the leaves.^11^ Compared with tea leaves, tea flowers have received less attention. Moreover, tea flowers have been regarded as a waste resource because the asexual propagation method has been widely applied in tea plant propagation, and they compete with tea leaves for nutrition and beverage.^12^ Chen et al. reported that 3000–12,000 kg of tea flowers is yielded annually per hectare of tea plantation.^12^ Tea flowers also contain tea catechins and caffeine in amounts that are comparable to those in tea leaves.^13^ It is critical to understand the factors that are likely to influence phytochemical extracts obtained from plants. However, the chemical components of plant extracts are affected by the species, genotype, organ type, environment, developmental phase, extraction methods, and other factors.^1^ Plant extracts have preeminent functional molecules such as saponins, different aromatic compounds, spermidine derivatives, etc.^12^ Due to the presence of these bioactive molecules, they have major health benefits including antioxidant activity, antiallergic activity, antidiabetic activity, and antitumor activity.^12^

Recently, the extracts of phenolic compounds extracted from tea flowers using different solvents including water,^14^ ethanol,^15^ and methanol^16^ have been studied through various extraction methods. However, there is no report in the literature on the optimization of bioactive compounds obtained from tea flowers using BG through UAE. Therefore, the present work studies the optimization of UAE used for the extraction of bioactive substances and antioxidant activities from tea flowers using response surface methodology (RSM). It also compares UAE and maceration as conventional extraction methods using BG and ethanol as conventional solvents for the extraction of bioactive compounds. It analyzes the antioxidant activities of these extracts and determines their phenolic compositions by liquid chromatography triple quadrupole tandem mass spectrometry (LC-QQQ). Finally, the cytotoxicity and cellular antioxidant activity of these tea flower extracts were evaluated. The researcher hopes that this study can provide a novel extraction method using a green solvent that could yield high bioactive ingredients with economic and environmental benefits for pharmaceutical and cosmetic applications.

## 2 Results and Discussion

### 2.1 Optimization of the Extraction Process Using RSM

BG had the highest total phenolic content (TPC) value compared to the other polyols including glycerol, propylene glycol, and water in our preliminary experiment (data not shown). Therefore, BG was selected as a green solvent for the optimization study. The optimization results of the extraction conditions for the active components and their antioxidant activities using RSM with Box–Behnken design (BBD) are reported in Table 1.

**Table 1:** BBD Presenting Predicted and Actual Values of Bioactive Compound Parameters and Antioxidant Assays

	amplitude (X_1_, %)		liquid–solid ratio (*X*_2_, mL/g)		solvent concentration (*X*_3_, %)		TPC (mg GAE/g)		TFC (mg QE/g)		TTC (mg TAE/g)		DPPH (mg TEAC/g)		ABTS (mg TEAC/g)		FRAP (mg FeSO_4_/g)	
run	coded	actual	coded	actual	coded	actual	predicted	actual	predicted	actual	predicted	actual	predicted	actual	predicted	actual	predicted	actual
1	–1	20	–1	20	0	80	28.48	27.22	157.50	131.73	34.34	32.88	67.95	70.91	106.16	111.21	55.38	48.66
2	1	40	–1	20	0	80	42.60	43.41	210.64	209.88	51.00	53.20	84.81	83.42	135.38	131.66	87.18	89.98
3	–1	20	1	30	0	80	36.82	36.01	174.14	174.90	40.74	38.54	51.57	52.94	103.02	106.73	74.36	71.56
4	1	40	1	30	0	80	44.98	46.23	244.80	270.59	49.36	50.83	85.39	82.41	131.36	126.30	95.56	102.27
5	–1	20	0	25	–1	60	45.10	44.59	210.54	221.51	57.98	57.26	70.51	69.05	137.23	128.94	92.46	95.23
6	1	40	0	25	–1	60	55.12	52.52	264.14	250.09	67.90	63.53	106.07	108.94	165.95	166.44	113.34	106.58
7	–1	20	0	25	1	100	8.98	11.58	27.12	41.17	9.84	14.21	25.13	22.23	30.09	29.61	21.58	28.34
8	1	40	0	25	1	100	21.24	21.76	97.32	86.34	25.20	25.92	40.25	41.70	58.93	67.21	53.70	50.94
9	0	30	–1	20	–1	60	46.11	47.87	215.78	230.58	59.65	61.82	90.32	88.80	149.12	152.35	90.54	94.51
10	0	30	1	30	–1	60	54.41	55.75	282.52	270.80	66.91	69.83	77.62	77.70	140.70	145.27	110.22	110.27
11	0	30	–1	20	1	100	14.05	12.72	82.00	93.73	19.11	16.19	29.92	29.82	37.20	32.61	31.28	31.24
12	0	30	1	30	1	100	16.47	14.70	66.06	51.26	16.61	14.43	26.82	28.33	38.46	35.23	38.96	35.00
13	0	30	0	25	0	80	46.85	49.38	293.50	304.75	56.32	59.42	105.09	107.65	142.78	144.82	110.64	109.34
14	0	30	0	25	0	80	46.85	45.34	293.50	306.02	56.32	52.81	105.09	105.65	142.78	148.68	110.64	117.29
15	0	30	0	25	0	80	46.85	48.94	293.50	303.36	56.32	59.81	105.09	101.06	142.78	145.74	110.64	108.13
16	0	30	0	25	0	80	46.85	44.28	293.50	287.68	56.32	57.22	105.09	109.23	142.78	143.28	110.64	111.77
17	0	30	0	25	0	80	46.85	45.46	293.50	258.69	56.32	51.05	105.09	100.33	142.78	138.27	110.64	104.48
18	0	30	0	25	0	80	46.85	47.73	293.50	300.50	56.32	57.61	105.09	106.60	142.78	135.89	110.64	112.87

#### 2.1.1 Fitting the Models

The analysis of variance of the response values of the TPC, total flavonoid content (TFC), total tannin content (TTC), 1,1-diphenyl-2-picrylhydrazil (DPPH), 2,2′-azino-bis-3-ethylbenzothiazoline-6-sulphonic acid (ABTS), and ferric reducing antioxidant power assay (FRAP) was carried out by using the R software program as shown in Table S1. The results showed that all the models had significant (*p* < 0.05) and non-significant lack of fit values (*p* ≥ 0.05). This indicates that all the models were suitable for the experimental design. Over 0.95 of the coefficients of determination (*R*^2^) of all the models demonstrated a good fit with the experimental data, and over 0.90 of the adjusted *R* squares (adj *R*^2^) of all the models showed that there were only few differences between the predicted and adjusted values. This signifies the reliability of the models.^17^ The regression coefficient values of these independent factors are presented in Table 2.

**Table 2:** Regression Analysis of all Responses in BBD^a^

	TPC				TFC				TTC			
parameter	estimated	std. Error	*t* value	Pr(>|*t*|)	estimated	std. Error	*t* value	Pr(>|*t*|)	estimated	std. Error	*t* value	Pr(>|*t*|)
Intercept	46.855	1.027	45.605	5.90 × 10^–11^***	293.500	9.524	30.816	1.34 × 10^–9^***	56.320	1.733	32.508	8.74 × 10^–10^***
*X*_1_	5.565	0.890	6.255	0.00024***	30.949	8.248	3.752	0.0056**	6.324	1.500	4.215	0.0029 **
*X*_2_	2.684	0.890	3.016	0.017*	12.704	8.248	1.540	0.162	1.193	1.500	0.795	0.450
*X*_3_	–17.496	0.890	–19.664	4.65 × 10^–8^***	–87.560	8.248	–10.616	5.43 × 10^–6^***	–22.711	1.500	–15.137	3.59 × 10^–7^***
*X*_1_:*X*_2_	–1.493	1.258	–1.186	0.270	4.385	11.665	0.376	0.717	–2.008	2.122	–0.946	0.372
*X*_1_:*X*_3_	0.563	1.258	0.447	0.667	4.148	11.665	0.356	0.731	1.360	2.122	0.641	0.539
*X*_2_:*X*_3_	–1.475	1.258	–1.172	0.275	–20.673	11.665	–1.772	0.114	–2.443	2.122	–1.151	0.283
*X*_1_^2^	–4.393	1.205	–3.646	0.0065**	–54.270	11.168	–4.859	0.0013**	–6.398	2.032	–3.149	0.014*
*X*_2_^2^	–4.245	1.205	–3.524	0.0078**	–42.455	11.168	–3.801	0.0052**	–6.060	2.032	–2.983	0.018*
*X*_3_^2^	–9.850	1.205	–8.176	3.73 × 10^–5^***	–89.453	11.168	–8.010	4.33 × 10^–5^***	–9.693	2.032	–4.771	0.0014**

	DPPH				ABTS				FRAP			
parameter	estimated	std. Error	*t* value	Pr(>|*t*|)	estimated	std. Error	*t* value	Pr(>|*t*|)	estimated	std. Error	*t* value	Pr(>|*t*|)
Intercept	105.090	1.528	68.758	2.23 × 10^–12^***	142.780	2.869	49.768	2.94 × 10^–11^***	110.647	2.663	41.549	1.24 × 10^–10^***
*X*_1_	12.668	1.324	9.571	1.18 × 10^–5^***	14.390	2.485	5.792	0.0004***	13.248	2.306	5.744	0.0004 ***
*X*_2_	–3.946	1.324	–2.981	0.018*	–1.788	2.485	–0.719	0.492	6.839	2.306	2.965	0.018 *
*X*_3_	–27.801	1.324	–21.004	2.77 × 10^–8^***	–53.543	2.485	–21.550	2.26 × 10^–8^***	–32.634	2.306	–14.150	6.05 × 10^–7^***
*X*_1_:*X*_2_	4.240	1.872	2.265	0.053	–0.220	3.514	–0.063	0.952	–2.653	3.262	–0.813	0.440
*X*_1_:*X*_3_	–5.105	1.872	–2.727	0.026*	0.025	3.514	0.007	0.994	2.813	3.262	0.862	0.414
*X*_2_:*X*_3_	2.403	1.872	1.284	0.235	2.425	3.514	0.690	0.510	–3.000	3.262	–0.920	0.385
*X*_1_^2^	–14.175	1.792	–7.909	4.74 × 10^–5^***	–8.560	3.364	–2.545	0.034*	–15.006	3.123	–4.805	0.0013**
*X*_2_^2^	–18.492	1.792	–10.318	6.71 × 10^–6^***	–15.245	3.364	–4.532	0.002**	–17.523	3.123	–5.612	0.0005***
*X*_3_^2^	–30.432	1.792	–16.981	1.47 × 10^–7^***	–36.170	3.364	–10.752	4.93 × 10^–6^***	–25.368	3.123	–8.124	3.91 × 10^–5^***

a Significant codes: “***” *p* < 0.001, “**” *p* < 0.01, and “*” *p* < 0.05.

#### 2.1.2 Effect of Variables on the TPC

The secondary polynomial equation for the TPC was obtained as follows
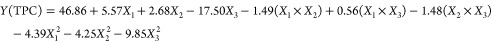
1

For the TPC, the linear terms of the amplitude and liquid–solid ratio had a positive and significant impact, suggesting that the TPC value increased with an increase in the amplitude and liquid–solid ratio (*p* < 0.05). Meanwhile, the solvent concentration had a significant and negative linear impact (*p* < 0.05), which means that the TPC decreased with increase in the solvent concentration. The interaction terms of all the variables did not have a significant impact on the TPC (*p* ≥ 0.05), as shown in Figure S1. However, all quadratic terms had a negative and significant impact on the TPC (*p* < 0.05). This shows that their response surface had a curvature, and the TPC value was at its highest under the optimal conditions of the variables, and a further increase in the variables would decrease the values of the TPC. Similarly, the effect of amplitude on the phenolic compounds obtained from the peel of *Punica granatum* var. Bhagwa through UAE was reported.^18^ The amplitude of the extraction efficiency may be due to the bubble collapse caused by high amplitude, exhibiting high shear forces and the initiation of microfractures or microcavities in the plant tissue, which led to the damage of the plant cell wall and release of the bioactive compounds into the solvents.^18^ The liquid–solid ratio theoretically influences the extraction of bioactive compounds.^19^ Several studies stated that the liquid–solid ratio is strongly related to the TPC.^20−22^ A prior investigation of coffee pulp extracted by the microwave-assisted extraction method with a water–ethanol mixture showed that the value of the TPC became higher with a rise in the liquid–solid ratio; however, it became lower with a rise in the solvent concentration.^21^ This may be because the concentration gradient increased as the solvent was added. The increase in the liquid–solid ratio enhances the mass transfer by increasing the amounts of components diffused in solvents. When the polyphenol content of the material is exhausted, the further increase in solvent amounts will not have any effect on the TPC extraction.^19^

#### 2.1.3 Effect of Variables on the TFC

The secondary polynomial equation for the TFC was obtained as follows
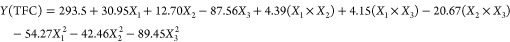
2

For the TFC, the linear terms of amplitude had a positive and significant impact, suggesting that an increase in amplitude could increase the TFC (*p* < 0.05). Meanwhile, the solvent concentration had a significant and negative linear impact (*p* < 0.05), which means that the TFC decreased with an increase in the solvent concentration. The interaction terms of all the variables did not have a significant impact on the TFC (*p* ≥ 0.05) as shown in Figure S1. However, all quadratic terms had a negative and significant impact on the TFC (*p* < 0.05), indicating that their response surface had a curvature, and the TFC value reached a maximum under the optimal conditions of the variables, and a further increase in variables would decrease the TFC values. An earlier study of flavonoids obtained from *Citrus aurantium* L. var. *amara* Engl. flowers via the ultrasound-assisted method supported the TFC findings in which the TFC increased as the solvent concentration increased up to 50%, after which it decreased with an increase in the solvent concentration.^23^ This could be explained by the miscibility of the solvent and the bioactive compounds. If the polarity of the extraction solvent is similar to that of the targeted bioactive compounds, the bioactive compounds can be extracted from plant cells easily.^23^

#### 2.1.4 Effect of Variables on the TTC

The secondary polynomial equation for the TTC was obtained as follows
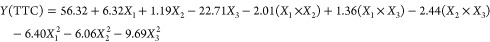
3

For the TTC, the linear terms of amplitude had a positive and significant impact, suggesting that an increase in amplitude could increase the TTC (*p* < 0.05). Meanwhile, the solvent concentration had a significant and negative linear impact (*p* < 0.05), indicating that the TTC decreased with an increase in the solvent concentration. The interaction terms of all variables did not have a significant impact on the TTC (*p* ≥ 0.05) as shown in Figure S1. However, all quadratic terms had a negative and significant impact on the TTC (*p* < 0.05). This shows that their response surface had a curvature, and the TTC value reached a maximum under the optimal conditions of the variables, and a further increase in the variables would decrease TTC values. A previous study of tannins obtained from *Cytinus hypocistis* (L.) via the UAE method reported that the highest TTC was obtained at a higher solvent concentration.^24^

#### 2.1.5 Effect of Variables on Antioxidant Activity

The secondary polynomial equations for DPPH, ABTS, and FRAP were as follows
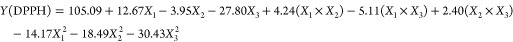
4
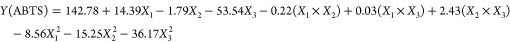
5
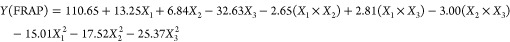
6

**Figure 1:**
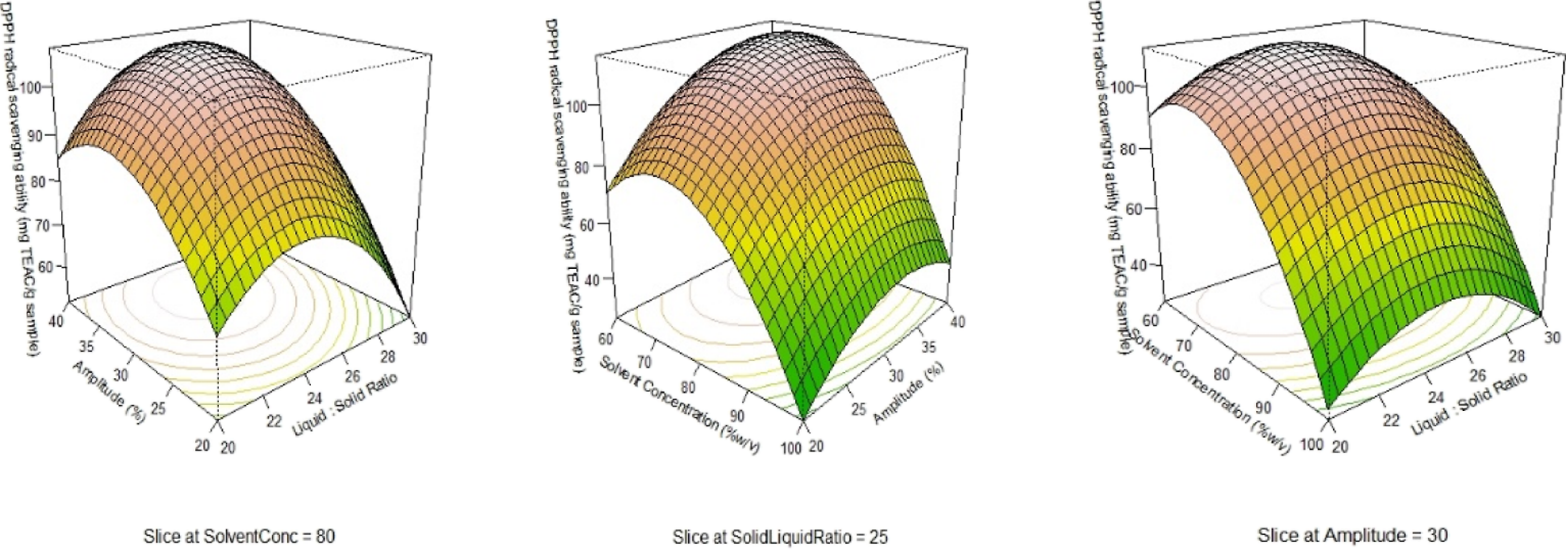
Three-dimensional response surface configurations for the impacts of the amplitude, liquid–solid ratio, and solvent concentration on DPPH.

Antioxidant activities were evaluated by DPPH, ABTS, and FRAP assays. In all the three assays, they shared similar patterns. The linear terms of amplitude had a positive and significant impact, and the linear terms of the solvent concentration and all quadratic terms had a negative and significant impact (*p* < 0.05). In addition, the linear term of the liquid–solid ratio had a negative and significant impact on DPPH and a positive significant impact on FRAP. The interaction terms of the amplitude and solvent concentration had a significant impact (*p* < 0.05) on DPPH. The three-dimensional response surface configurations for the effects of the amplitude, liquid–solid ratio, and solvent concentration on DPPH are presented in Figure 1. With an increase in amplitude from 20 to 30%, the values of their antioxidant activities increased linearly. However, as the amplitude increased from 30 to 40%, their values did not increase significantly. Similar findings for DPPH were reported in a previous study of phenolic compounds obtained from the peel of *P. granatum* var. Bhagwa via UAE.^18^ Similarly, when the liquid–solid ratio increased from 20 to 25%, their values increased linearly. However, when the liquid–solid ratio increased from 25 to 30%, it did not change their values significantly. On the contrary, their values decreased linearly with an increase in the solvent concentration from 60 to 100%. In a prior investigation of phenolics extracted from *Rheum moorcroftianum* rhizomes via the ultrasound-assisted method, the capacity of DPPH and ABTS increased with a rise in the liquid–solid ratio until it met its dissolution equilibrium; however, with a subsequent rise in the liquid–solid ratio, it had a downward trend.^25^ In addition, similar results for FRAP were reported in a recent investigation of *Lithocarpus polystachyus* Rehd extracted by microwave-assisted extraction methods.^26^

### 2.2 Validation of the Predicted Value

Under the optimal conditions of an amplitude of 29.15%, a liquid–solid ratio of 23.65 mL/g, and a solvent concentration of 63.45%, the values of all responses experimentally obtained are presented in Table 3, and all these values do not show a significant difference from the predicted values of the responses (*p* ≥ 0.05). These findings verified the validity of the response model.

**Table 3:** Optimal Conditions, Predicted Values, and Experimental Values^a^

condition	amplitude (%)	liquid–solid ratio	solvent concentration (%)	TPC (mg GAE/g sample)	TFC (mg QE/g sample)	TTC (mg TAE/g sample)	DPPH (mg TEAC/g sample)	ABTS (mg TEAC/g sample)	FRAP (mg FeSO_4_/g sample)
Predicted	29.15	23.65 mL/g	63.45	52.72	290.93	66.63	106.07	160.94	115.39
Actual	29.15	23.65 mL/g	63.45	54.00 ± 1.19^ns^	291.47 ± 3.34^ns^	65.37 ± 1.78^ns^	106.45 ± 1.21^ns^	163.58 ± 2.76^ns^	121.31 ± 4.75^ns^

a The response values are presented as mean ± standard deviation (*n* = 3). ns means that the values within the same column were not significantly different (*p* ≥ 0.05).

### 2.3 Comparison of UAE with the Conventional Method and Solvent

Due to their safety, relatively low extraction cost, easy storage, and simple scalability with less time and energy consumption, the plant-derived versatile products are in high demand for food, pharmaceutical, and cosmetic applications.^27^ To achieve this goal, the conventional methods were compared by using conventional solvents for the extraction of bioactive compounds. The conventional method and conventional solvent used in this study are maceration and ethanol, respectively, because they are well known for the extraction of bioactive compounds in cosmetic and pharmaceutical industries.^28^ In this study, a dried tea flower was extracted through BG-UAE, EtOH-UAE, maceration with BG (BG-MAR), and maceration with EtOH (EtOH-MAR) using the ideal parameters of the response models. The results with significant levels are shown in Table 4. BG-UAE demonstrated the highest value in all responses, except the FRAP value, while BG-MAR had the highest value in FRAP activity and the second highest value in all the remaining responses. However, EtOH-MAR demonstrated the least value in all the responses except the TPC, and EtOH-UAE produced the least TPC value. These findings showed that BG, rather than ethanol, produced statistically larger yields of bioactive chemicals with antioxidant activities from the tea flowers by UAE or maceration. Using the same extraction solvent, UAE produced greater results for all responses, except FRAP value, and took less time compared to the maceration method. In addition, compared to a previous study, the TPC and TFC of the tea flower extracted by BG-UAE were greater than those extracted by maceration with methanol.^16^

**Table 4:** Comparative Study between UAE and Maceration Using BG and Ethanol^a^

condition	amplitude (%)	liquid–solid ratio (mL/g)	solvent concentration (%)	time	TPC(mg GAE/g sample)	TFC(mg QE/g sample)	TTC(mg TAE/g sample)	DPPH(mg TEAC/g sample)	ABTS(mg TEAC/g sample)	FRAP(mg FeSO_4_/g sample)
BG-UAE	29.15	23.65	63.45	5 min	54.00 ± 1.19^a^	291.47 ± 3.34^a^	65.37 ± 1.78^a^	106.45 ± 1.21^a^	163.58 ± 2.76^a^	121.31 ± 4.75^a^
EtOH-UAE	29.15	23.65	63.45	5 min	50.67 ± 0.50^b^	280.39 ± 2.10^b^	55.48 ± 1.20^c^	89.27 ± 2.37^bc^	146.14 ± 7.31^bc^	111.44 ± 2.42^b^
BG-MAR		23.65	63.45	24 h	53.02 ± 1.39^a^	288.08 ± 3.85^a^	61.36 ± 0.34^b^	90.80 ± 2.65^b^	154.45 ± 4.82^ab^	125.03 ± 1.54^a^
EtOH-MAR		23.65	63.45	24 h	50.74 ± 0.79^b^	278.88 ± 3.22^b^	53.83 ± 3.28^c^	86.03 ± 2.26^c^	143.09 ± 6.89^c^	111.32 ± 2.39^b^

a The response values are presented as mean ± standard deviation (*n* = 3). Different superscript letters in the same column indicate that the values were statistically and significantly different (*p* < *0.05*).

### 2.4 Identification and Quantification of Phenolic Compounds by LC-QQQ

The active components of tea flower extracts were identified and quantified by LC-QQQ. The results of the bioactive substances of tea flower extracts are shown in Table 5. The most abundant phenolic and alkaloid compounds in both tea flower extracts are epigallocatechin gallate (EGCG), caffeine, and other catechins. A previous study reported that the ethanolic extracts of tea flowers contained various catechins, gallates, and caffeine as major components.^13^ Except for catechin gallate (CG), EGCG, gallocatechin (GC), and kaempferol, which were found in higher concentrations in the EtOH-UAE extract, the BG-UAE extract in this study had the highest amounts of the bioactive components. Subsequently, BG extracts exhibited higher antioxidant activities compared to the ethanolic extract. The variations in these compounds may be due to the differences in the bioactive activities of the tea flower extracts. Several marked amounts from the literature mainly emphasize the phenolic compounds of edible plants due to the health benefits of polyphenols.^1,2^ The bioactive components in tea flowers offer various benefits for the skin. They might have an anti-inflammatory, anti-allergic, and antioxidant effect on our skin.^29^ Additionally, they can inhibit tyrosinase activity and melanin synthesis.^29^ This implies that tea flower extracts may offer these potential advantages.

**Table 5:** Yield Value and %Increase of the Phenolic Compositions and Caffeine Content in BG-UAE and EtOH-UAE Extracts^a^

yield value (mg/1 g of the sample)			
bioactive compounds	BG-UAE	EtOH-UAE	% change compared to EtOH-UAE
gallic acid	N.A.	N.A.	1.285
caffeine	1.997 ± 0.073^ns^	1.935 ± 0.081	3.197
catechin	0.081 ± 0.004^ns^	0.077 ± 0.005	4.655
CG	0.797 ± 0.005*	0.937 ± 0.041	–14.935
epicatechin (EC)	0.206 ± 0.002*	0.184 ± 0.003	12.18
EGCG	2.236 ± 0.232*	2.744 ± 0.213	–18.517
epigallocatechin (EGC)	0.191 ± 0.002*	0.156 ± 0.003	22.597
gallocatechin gallate (GCG)	0.016 ± 0.001^ns^	0.015 ± 0.001	6.393
GC	0.742 ± 0.104^ns^	0.893 ± 0.061	–16.884
*p*-coumaric acid	N.A.	N.A.	2.962
protocatechuic acid	N.A.	N.A.	5.768
ferulic acid	N.A.	N.A.	26.576
4-hydroxybenzoic acid	N.A.	N.A.	2.854
kaempferol	N.A.	N.A.	–6.609
naringenin	N.A.	N.A.	29.282
theobromine	N.A.	N.A.	2.060

a Values are represented as mean ± SD (*n* = 3). * indicates that the values in the same row are significantly different (*p* < 0.05). ns indicates that the values in the same row are not significantly different (*p* ≥ 0.05). N.A. stands for non-availability of bioactive standards. The percent increase of bioactive components was estimated using the difference between their peak areas in BG-UAE and EtOH-UAE.

### 2.5 Cell Culture

#### 2.5.1 Cytotoxicity Assay

**Figure 2:**
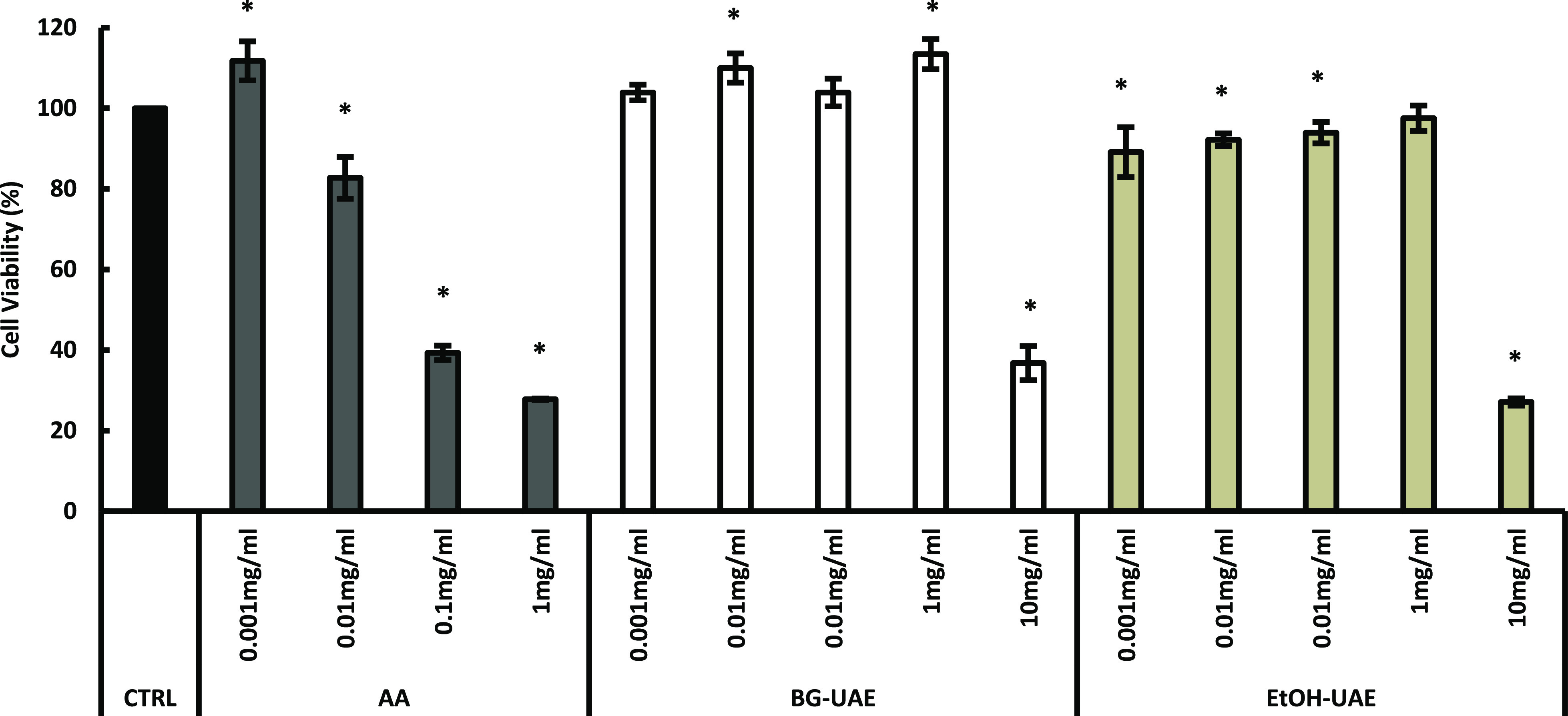
Results of cytotoxicity of different concentrations of AA, BG-UAE, and EtOH-UAE. * points out that the values were significantly different from those of the control (*p* < 0.05). CTRL: control.

Cytotoxicity was evaluated using various concentrations of ascorbic acid (AA) and tea flower extracts to determine their maximum non-toxic concentration. Figure 2 shows the results of the cytotoxicity assay of AA and tea flower extracts. The viability of cells treated with 0.001 and 0.01 mg/mL AA was higher than 80% cell viability; thus, it could be concluded that these concentrations were non-cytotoxic concentrations.^30^ Wu et al. stated that higher concentrations of AA could be cytotoxic to cells as they enhance cell apoptosis by inducing metabolic stress.^31^ Apart from the cells treated with a concentration of 10 mg/mL of both BG-UAE and EtOH-UAE extracts, which showed cell viability below 80%, cells treated with the remaining concentrations of 0.001, 0.01, 0.1, and 1 mg/mL of both extract samples expressed cell viability higher than 80%. Thus, these concentrations were considered to be non-cytotoxic. Upon comparing the cell viability between tea flower extracts at the same concentration, cells treated with BG-UAE showed greater cell viability than those treated with EtOH-UAE at all concentrations. This is explained by a former investigation of cytotoxicity on cosmetic materials to mouse fibroblasts by 3-(4,5-dimethylthiazol-2-yl)-2,5-diphenyltetrazolium bromide (MTT) assay which reported that BG was less toxic than propylene glycol, glycerin, and other moisturizers.^32^ A previous study of the cytotoxicity of aqueous-ethanol tea flower extracts to B16-F10 melanoma cells reported that tea flower extracts exhibited dose-dependent cytotoxicity.^33^

#### 2.5.2 Cellular Antioxidant Assay

**Figure 3:**
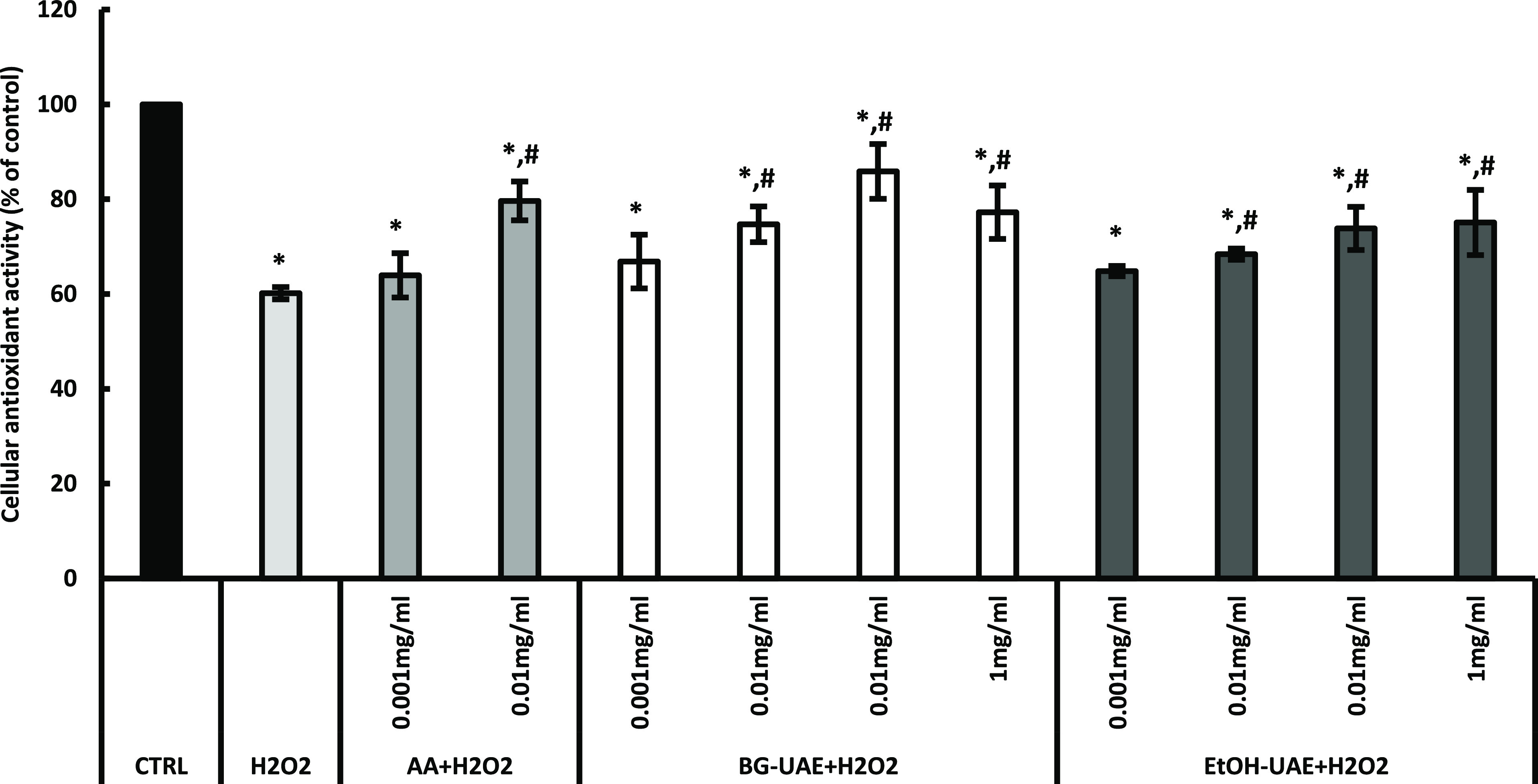
Cellular antioxidant capacity of AA, BG-UAE, and EtOH-UAE. * points out that the values were significantly different from those of the control (*p* < 0.05); # points out that the values were significantly different from those of the H_2_O_2_ group (*p* < 0.05). CTRL: control.

The cellular antioxidant activities of the tea flower extracts were evaluated by treating cells that induce oxidative stress with H_2_O_2_, followed by MTT assay.^34^Figure 3 presents the results of the cellular antioxidant effect of the tea flower extracts on 400 μM H_2_O_2_-induced oxidative stress in the NIH/3T3 fibroblasts. In comparison to the control, a significant reduction of the cell viability to 60.17 ± 1.28% was found in the cells treated with H_2_O_2_ (*p* < 0.05). However, when the cells were treated with AA at 0.01 mg/mL before treating them with H_2_O_2_, the viability of the cells was significantly increased to 79.64 ± 4.09% compared to that of the cells treated with H_2_O_2_ (*p* < 0.05). For tea flower extracts, apart from the cells treated with the 0.001 mg/mL concentration of both extracts, the cells treated with the remaining concentrations of the BG-UAE extract and EtOH-UAE extract showed a statistically significant increase in cell viability compared to those treated with H_2_O_2_ (*p* < 0.05). Thus, it could be concluded that both tea flower extracts showed dose-dependent cellular antioxidant effects. In the comparison of the antioxidant capacity between these two extracts at the same concentration, the BG-UAE extract demonstrated greater cell viability compared to the EtOH-UAE extract. In both extracts, various types of catechins and caffeine were predominantly detected. Different catechins exhibited different antioxidant activities based on their chemical structure.^35^ According to Hong et al., EGC was the most active compound among catechins followed by EGCG, GA, and EC.^36^ In this study, the BG-UAE extract had higher cellular antioxidant activity because it contained a higher content of bioactive components including EGC, caffeine, EC, and other compounds compared to the EtOH-UAE extract. A previous study of aqueous and ethanol extraction of tea flowers reported that both extracts exhibited strong cellular antioxidant effects in lipopolysaccharide-induced RAW 264.7 cells.^13^

## 3 Conclusions

This study optimizes the UAE method used for extracting tea flowers with BG as the solvent. The percent of amplitude and the solvent concentration were found to be the factors affecting the extraction of tea flower samples in all responses with the latter being the strongest factor among the three independent variables. Catechins and caffeine were the predominant bioactive compounds in BG-UAE which exhibited both in vitro and cellular antioxidant activities as compared to those in EtOH-UAE. BG-UAE can be considered a novel green bioactive extraction method that can yield more bioactive chemicals with higher antioxidant activities. Furthermore, BG-UAE reduced the downstream process including the elimination of harmful solvents used during the extraction, resulting in reduced extraction time, lower energy consumption, easier scale-up, and cost-effective strategies. Hence, UAE of tea flowers using BG could be suitable for food, pharmaceutical, and cosmetic application as bioactive substances to be utilized as functional molecules in medicinal and cosmetic uses. The other biological activities of this extract would be further evaluated, and the clinical studies of this extract should be studied to confirm its biological activities in human volunteers.

## 4 Methods

### 4.1 Tea Flower Sample Preparation

The fresh tea flower was kindly provided by tea plantation 101, Doi Mae Salong, Chaing Rai, Thailand. The fresh tea flower sample was dried in a tray dryer at 60 °C for 48 h. The tea flower was then ground to a fine powder using a mechanical grinder and kept at room temperature until use.

### 4.2 Optimization of the Extraction Method

An optimization experiment was performed using the RSM with a BBD for the extraction of active compounds from the tea flower. Variables including the amplitude (*X*_1_, %), liquid–solid ratio (*X*_2_: mL/g), and solvent concentration (*X*_3_, %) were selected and kept at three levels (−1, 0, and +1) as presented in Table S3. The experimental results of BBD were used to determine the optimal conditions that might produce the highest levels of bioactive compounds and antioxidant properties of the tea flower. The second-order polynomial equation for each response variable was evaluated as follows
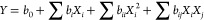
7where *X*_*i*_ and X_*j*_ values are independent variables; *b*_0_ is the intercept; and *b*_*i*_, *b*_*ii*_, and *b*_*ij*_ are the regression coefficients for linear, quadratic, and interaction terms, respectively.

### 4.3 Ultrasonic-Assisted Extraction of Tea Flowers

The protocol outlined by Wen et al.^37^ was slightly modified to extract the bioactive components from the tea flower. An ultrasonic processor (VCX 130, Vibra cell, Sonics, USA) with a 6 mm probe was used to extract the tea flowers. During the extraction cycle, the probe was placed 2 cm into the extraction solvent. 1 g of the powdered tea flowers was mixed with different BG concentrations and different liquid–solid ratios and extracted at different amplitudes as shown in Table 1 for an extraction duration of 5 min at room temperature. The resulting mixture was centrifuged using 2490*g* at 4 °C for 15 min.

### 4.4 Bioactive Compound Analysis

The TPC, TFC, and TTC were determined according to the methods outlined by Myo and Khat-udomkiri (2022).^34^ The results were expressed as mg of the gallic acid equivalent (GAE) per gram of the sample for the TPC, mg of the quercetin equivalent (QE) per gram of the sample for the TFC, and mg of the tannic acid equivalent (TAE) per gram of the sample for the TTC.

### 4.5 Antioxidant Activities

DPPH radical scavenging assay, ABTS radical scavenging assay, and FRAP were evaluated according to the protocols stated by Myo et al.^38^ The values were expressed as mg of Trolox equivalent antioxidant capacity (TEAC) per gram of the sample for DPPH and ABTS assays and mg of FeSO_4_ per gram of the sample for FRAP assay.

### 4.6 Validation of the Predicted Value

By the response model, a validation experiment was assessed utilizing the optimal BBD extraction parameters to verify the accuracy of the response model.

### 4.7 Comparison of UAE with the Conventional Method and Conventional Solvent

Different solvents (BG or EtOH) were used in a comparison study between the UAE and a conventional approach (maceration).

#### 4.7.1 UAE with BG or EtOH

BG-UAE and EtOH-UAE were carried out by the above-mentioned procedure under optimal extraction conditions determined by BBD. After the centrifugation of the mixtures, the supernatants collected were kept for the analysis of their bioactive compounds and antioxidant activities.

#### 4.7.2 Maceration with BG or EtOH

Zhao et al.’s^39^ method with slight modifications was used for the maceration of the tea flower with BG and EtOH. Briefly, 1 g of the tea flower was mixed with 63.45% w/v of BG or EtOH at a liquid–solid ratio of 23.65 mL/g. An incubated shaker was used to macerate the mixtures at a speed of 100 rpm and 25 °C for 24 h. After the centrifugation of the resultant mixtures, the supernatants collected were kept for the analysis of the bioactive compounds and their antioxidant activities.

### 4.8 Identification and Quantification of the Phenolic Compositions and Caffeine Content by LC-QQQ

A method outlined by Saftic et al.^40^ was slightly modified to identify and quantify the active ingredients in tea flowers. A Nexera X2 UHPLC system (Shimadzu, Kyoto, Japan) joined with an LCMS-8060 triple quadrupole mass spectrometer (Shimadzu, Kyoto, Japan) was operated in both positive and negative electrospray ionization modes. Chromatographic separation was evaluated on a C18 reversed-phase Avantor ACE Excel C18-PFP (100 mm × 2.1 mm, 1.7 μm) analytical column. The results of LC-QQQ were evaluated using LabSolutions software (Shimadzu, Kyoto, Japan). LC-QQQ parameters for active components of the tea flower are presented in Table S2. By comparing sample peak areas to those of bioactive chemical standards, active components were quantified, and the results were reported as mg per gram of the sample.

### 4.9 Cell Culture

The NIH/3T3 fibroblasts (ATCC CRL-1658TM) were obtained from the American Type Culture Collection (Manassas, VA, USA). The cells were grown at 37 °C in a humidified incubator with 5% CO_2_ (Binder, model CB210, Germany) using Dulbecco’s modified Eagle’s medium (DMEM) plus 10% fetal bovine serum (FBS) and 1% penicillin/streptomycin solution. The cell passages from 16 to 23 were utilized in these experiments.

### 4.10 Cytotoxicity Assay

A protocol stated by Park et al.^41^ was used to perform the MTT assay to assess the mitochondrial functionality of the NIH/3T3 cells. NIH/3T3 cells were treated with either AA, BG-UAE, or EtOH-UAE extracts, while DMEM without the FBS supplement was used as the control for 24 h. MTT is reduced by mitochondrial dehydrogenases, producing a purple formazan product. Dimethyl sulfoxide solution was used to dissolve formazan crystals. The dissolved formazan can be quantified spectrophotometrically and is proportionate to the number of viable cells. The absorbance was measured at 570 nm. Cell viability (%) was calculated as follows
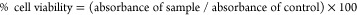


### 4.11 Cellular Antioxidant Activity

The cellular antioxidant assay was performed using the method outlined by Myo and Khat-udomkiri (2022).^34^ NIH/3T3 cells were treated with either AA, BG-UAE, or EtOH-UAE extracts, while DMEM without the FBS supplement was used as the control for 24 hours followed by H_2_O_2_ treatment. Cell viability (%) was quantified by MTT assay and calculated as
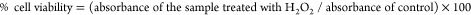


### 4.12 Statistical Analysis

The experiments in this study were assessed in triplicate. Using R software and the RSM package, the statistical analysis of BBD in RSM was done. During the validation phase, the difference between the actual value and the predicted value was analyzed using a one-sample *t*-test. The comparison between extraction techniques in terms of bioactive substances and antioxidant activity was assessed by using one-way analysis of variance (ANOVA). The least significant difference (LSD 0.05) test was used to analyze the pairwise comparison between the groups. Mean ± standard deviation was used to express all data.

## Supporting Information Available

Three-dimensional response surface configurations for the impacts of the amplitude, liquid–solid ratio, and solvent concentration on bioactive compounds and antioxidant activities; ANOVA of all responses in BBD; LC-QQQ parameters of bioactive compounds for analysis of the *Camellia sinensis* flower extract; and factors and their levels for BBD (PDF)

## Abbreviations

ABTS: 2,2′-azino-bis-3-ethylbenzothiazoline-6-sulphonic acid
BBD: Box–Behnken design
BG: butylene glycol
BG-MAR: maceration with butylene glycol
BG-UAE: ultrasound-assisted extraction with butylene glycol
DPPH: 1,1-diphenyl-2-picrylhydrazil
EtOH: ethanol
EtOH-UAE: ultrasound-assisted extraction with ethanol
EtOH-MAR: maceration with ethanol
FDA: Food and Drug Administration
FRAP: ferric reducing antioxidant power
GAE: gallic acid equivalent
GRAS: generally recognized as safe
H_2_O_2_: hydrogen peroxide
LC-QQQ: liquid chromatography triple quadrupole tandem mass spectrometry
MTT: 3-(4,5-dimethylthiazol-2-yl)-2,5-diphenyltetrazolium bromide
QQQ: triple quadrupole mass spectrometer
QE: quercetin equivalent
RSM: response surface methodology
TAE: tannic acid equivalent
TEAC: Trolox equivalent antioxidant capacity
TF: tea flower
TFC: total flavonoid content
TPC: total phenolic content
TTC: total tannin content
UAE: ultrasound-assisted extraction
