# Supplementary material for: Butylene Glycol Used as a Sustainable Solvent for Extracting Bioactive Compounds from Camellia sinensis Flowers with Ultrasound-Assisted Extraction
Source: ACS Omega. 2023 Jan 25;8(5):4976–87. doi: 10.1021/acsomega.2c07481 (PMC9909804; doi:10.1021/acsomega.2c07481)
Supplement: Supplementary file 1 — ao2c07481_si_001.pdf [file ao2c07481_si_001.pdf]

Supporting Information

Butylene glycol used as a sustainable solvent for extracting bioactive compounds from *Camillia sinensis* flowers with ultrasound-assisted extraction

Hla Myo [a], *Nara Yaowiwat* [a], *Pawin Pongkorpsakol* [b], *Chanat Aonbangkhen* [c,d], *and Nuntawat Khat-udomkiri* [a,*]

[a] School of Cosmetic Science, Mae Fah Laung University, Thailand. Emails: 6351701003@lamduan.mfu.ac.th (HM); nara.nan@mfu.ac.th (NY); nuntawat.kha@mfu.ac.th (NK)

[b] Princess Srisavangavadhana College of Medicine, Chulabhorn Royal Academy, Bangkok, Thailand. Emails: pawin.pon@cra.ac.th (PP)

[c] Department of Chemistry, Faculty of Science, Chulalongkorn University, Bangkok, Thailand. Emails: Chanat.A@chula.ac.th (CA)

[d] Center of Excellence in Natural Products Chemistry (CENP), Department of Chemistry, Faculty of Science, Chulalongkorn University, Bangkok, Thailand. Emails: Chanat.A@chula.ac.th (CA)

ASSOCIATED CONTENT

Supporting Information

a

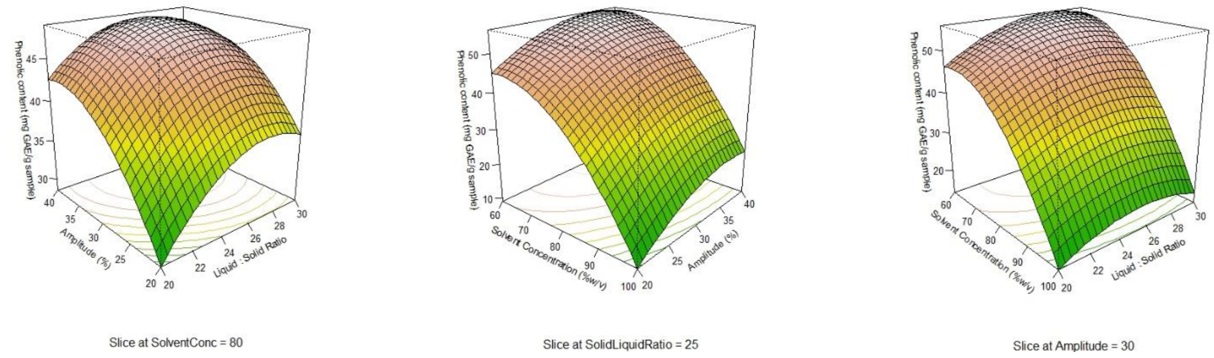

b

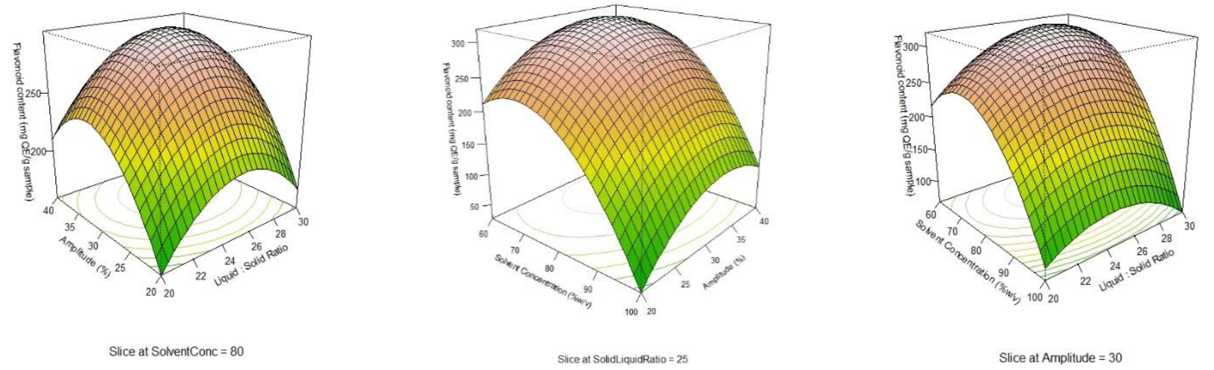

c

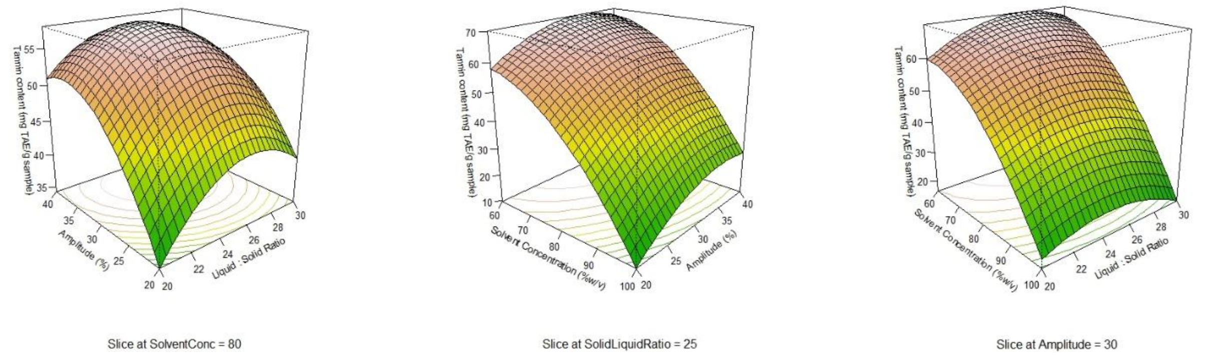

d

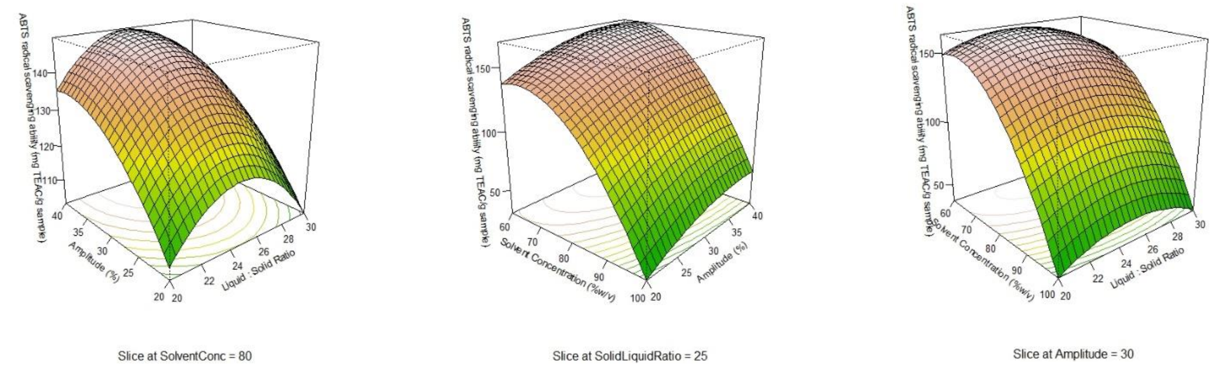

e

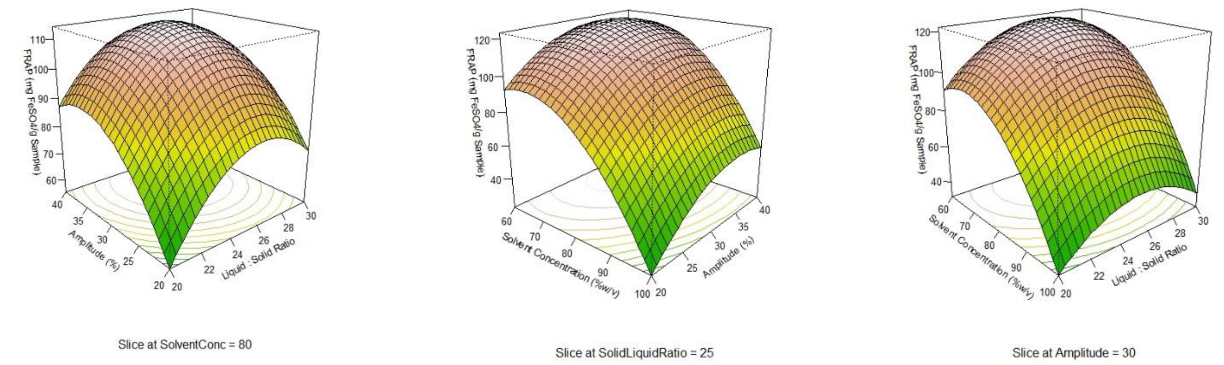

Figure S1 The three-dimensional response surface configurations for the impacts of amplitude, liquid-solid ratio, and solvent concentration on TPC (a), TFC (b), TTC (c), ABTS (d), and FRAP (e).

Table S1. Analysis of variance of all responses in BBD

Response	Source	Sum Sq	Df	Mean Sq	F value	Pr (>F)
	Model	3462.45	9	384.72	60.74	2.09E-06
	Linear	2754.32	3	918.11	144.96	2.60E-07
	Interaction	18.88	3	6.29	0.99	0.444
	Quadratic	689.25	3	229.75	36.28	5.26E-05
TPC	Residuals	50.67	8	6.33		
	Lack of fit	28.31	3	9.44	2.11	0.218
	Pure error	22.36	5	4.47		
	Total	3513.12	17			
	R square	0.9856				
	Adj R square	0.9694				
	Model	138546	9	15394.0	28.28	3.98E-05
	Linear	70288	3	23429.2	43.05	2.78E-05
	Interaction	1855	3	618.4	1.14	0.391
	Quadratic	66403	3	22134.3	40.67	3.44E-05
TFC	Residuals	4354	8	544.3		
	Lack of fit	2679	3	893.0	2.67	0.159
	Pure error	1675	5	335.0		
	Total	142900	17			
	R square	0.9695				
	Adj R square	0.9352				
	Model	5409.5	9	601.06	33.37	2.12E-05
	Linear	4457.7	3	1485.9	82.51	2.34E-06
	Interaction	47.4	3	15.79	0.88	0.492
	Quadratic	904.4	3	301.48	16.74	8.30E-04

TTC	Residuals	144.1	8	18.01		
	Lack of fit	79.7	3	26.57	2.06	0.223
	Pure error	64.4	5	12.87		
	Total	5553.6	17			
	R square	0.9741				
	Adj R square	0.9449				
	Model	15436.3	9	1715.14	122.4	1.33E-07
	Linear	7591.6	3	2530.53	180.55	1.10E-07
	Interaction	199.2	3	66.41	4.73	0.035
	Quadratic	7645.5	3	2548.49	181.84	1.07E-07
DPPH	Residuals	112.1	8	14.02		
	Lack of fit	46.9	3	15.65	1.2	0.39932
	Pure error	65.2	5	13.04		
	Total	15548.4	17			
	R square	0.9928				
	Adj R square	0.9847				
	Model	32694.7	9	3632.74	73.56	9.88E-07
	Linear	24616.5	3	8205.5	166.16	1.52E-07
	Interaction	23.7	3	7.9	0.16	0.920
	Quadratic	8054.5	3	2684.8	54.37	1.15E-05
ABTS	Residuals	395.1	8	49.4		
	Lack of fit	279.3	3	93.1	4.02	0.084
	Pure error	115.8	5	23.2		
	Total	33089.8	17			
	R square	0.9881				
	Adj R square	0.9746				
	Model	16586.6	9	1842.96	43.31	7.77E-06

	Linear	10297.8	3	3432.6	80.67	2.55E-06
	Interaction	95.8	3	31.9	0.75	0.552
	Quadratic	6193.0	3	2064.3	48.52	1.78E-05
FRAP	Residuals	340.4	8	42.6		
	Lack of fit	244.0	3	81.3	4.22	0.078
	Pure error	96.4	5	19.3		
	Total	16927	17			
	R square	0.9799				
	Adj R square	0.9573				

Table S2. LC-QQQ parameters of bioactive compounds for analysis of *Camillia sinensis* flower extract

Bioactive Components	Retention Time (RT, min)	Ionization Mode of the Analytes	Precursor Ion (m/z, $[M-H]^-$)	Product Ion (m/z)
Gallic acid	1.639	Negative	169.10	125.20
Caffeine	3.589	Positive	195.00	138.15, 110.10
Catechin	3.955	Negative	289.25	205.20, 245.20
Catechin Gallate (CG)	5.630	Negative	441.20	169.20
Epicatechin (EC)	4.766	Negative	289.00	245.30, 203.10, 109.20
Epigallocatechin Gallate (EGCG)	5.059	Negative	457.00	169.20, 305.20
Epigallocatechin (EGC)	3.508	Negative	304.80	125.10, 219.10
Gallocatechin Gallate (GCG)	5.312	Negative	456.85	169.20, 305.20
Gallocatechin (GC)	2.349	Negative	304.95	125.20, 219.20
p-coumaric acid	5.495	Negative	162.85	119.00
Protocatechuic acid	2.627	Negative	153.25	109.05
Ferulic acid	5.827	Negative	193.00	134.10
4-hydroxybenzoic acid	6.008	Negative	137.05	92.95
Kaempferol	5.780	Negative	285.00	143.05,159.10,187.20
Naringenin	6.232	Negative	271.05	106.95,119.05
Theobromine	1.763	Positive	181.10	163.10,138.10,110.05

Table S3. Factors and their levels for Box–Behnken design.

Factors	Label	Levels		
		-1	0	1
Amplitude (%)	X_1	20	30	40
Liquid-solid ratio (mL/g)	X_2	20	25	30
Solvent concentration (%)	X_3	60	80	100
